# Pathogenic Characteristics of Five Different Lineage of Korean PRRSV-2 Isolates (NADC30-Like, VR2332-Like, LKA, LKB, and LKC)

**DOI:** 10.1155/2024/1618472

**Published:** 2024-10-25

**Authors:** Chang-Gi Jeong, Seung-Chai Kim, Simin Lee, Hwan-Ju Kim, Sameer ul Salam Mattoo, Salik Nazki, Amina Khatun, Go-Eun Shin, Myeon-Sik Yang, Hye-Young Jeoung, Kyoung-Ki Lee, Jae-Ku Oem, Sang-Myeong Lee, Bumseok Kim, Gayeon Won, Won-Il Kim

**Affiliations:** ^1^College of Veterinary Medicine, Jeonbuk National University, Iksan 54596, Republic of Korea; ^2^Biosafety Research Institute, Iksan 54596, Republic of Korea; ^3^Melbourne Veterinary School, Faculty of Science, The University of Melbourne, Parkville 3052, Victoria, Australia; ^4^Division of Biotechnology, Jeonbuk National University, Iksan 54596, Republic of Korea; ^5^Division of Veterinary Public Health, Faculty of Veterinary Sciences and Animal Husbandry, SKUAST-Kashmir, Srinagar, India; ^6^Department of Pathology, Faculty of Animal Science and Veterinary Medicine, Sher-e-Bangla Agricultural University, Sher-e-Bangla Nagar, Dhaka 1207, Bangladesh; ^7^Animal and Plant Quarantine Agency, 177 Hyeoksin 8-ro, Gimcheon 39660, Republic of Korea; ^8^Department of Companion and Laboratory Animal Science, Kongju National University, Yesan-eup, Chungcheongnam-do, Gongju 32439, Republic of Korea; ^9^College of Veterinary Medicine, Chungbuk National University, Chungju, Republic of Korea

## Abstract

Porcine reproductive and respiratory syndrome virus (PRRSV) is a significant pathogen in the worldwide swine industry. The virus shows high genetic variation coupled with a broad range of virulence in pigs. Although multiple lineages of the virus have been prevalent throughout in Korea, the characteristics of lineage-wise pathogenicity are largely unknown. Therefore, this study was designed to analyze and compare the pathogenicity of 11 representative Korean PRRSV-2 isolates selected from PRRSV-2 lineages circulating in Korea, NADC30-like, VR2332-like, and three nation-specific lineages (lineage KOR A (LKA), lineage KOR B (LKB), and lineage KOR C (LKC)), which have been continuously prevalent in the nation. Eleven groups of pigs were experimentally infected with one Korean PRRSV-2 isolate through four consecutive animal experiments. Body weight and body temperature were recorded during each 4-week challenge experiment period, and virological, serological, and histopathological tests were performed on the collected samples. The data from the animal experiments were integrated into two indicators—excretion and clinical signs—through correlation and principal component analysis (PCA). Meta-analysis was used to compare PRRSV-2 isolates using each indicator. Based on these analyses, while L1C viruses used in this study (JB15-N-P31-GB and JB15-N-PJ73-GN, similar to NADC30-like strains) exhibited low or moderate levels of excretion and clinical signs, lineage 5 (L5) or modified live vaccine (MLV)-variant strains exhibited high levels of excretion compared to other PRRSV-2 isolates. However, the L5 variants all caused mild clinical signs, except for JB15-N-PJ4-GN, which showed the 4th highest clinical sign indicator. Among the Korean lineages (LKA, LKB, and LKC), two LKB strains (GGYC45 and JB15-N-PJ10-GN) were the most virulent as they showed the highest mortality after the challenge. On the other hand, the LKA and LKC viruses displayed lower excretion indicators than L5 strains, but they had higher-ranked clinical sign indicators than low-virulence L5 MLV variants. In conclusion, PRRSV prevalent in Korea has diverse excretion and clinical characteristics, and certain lineage is highly pathogenic. These results will offer useful insights to prevent spread of PRRSV and improve the efficacy of vaccines in the future.

## 1. Introduction

Porcine reproductive and respiratory syndrome (PRRS) was first recognized in the United States in 1987 and is now considered one of the most threatening diseases to the global swine industry [1]. PRRS is a disease that causes respiratory distress in pigs of all ages and severe reproductive failure in breeding sows [2]. The estimated annual loss to the U.S. swine industry associated with PRRS is approximately $664 million [3]. The causative agent of this disease is the PRRS virus (PRRSV), which is classified as a *Betaarterivirus* by the International Committee on Taxonomy of Viruses and belongs to the order *Nidovirales*, *Arteriviridae* family [4, 5]. PRRSV is an enveloped virus that has an approximately 15 kb, nonsegmented, single-stranded, positive-sense RNA genome that encodes at least 11 open reading frames (ORFs), a 5′ and 3′ untranslated region (UTR), and a 3′ poly (A) tail [4, 6, 7]. In addition, PRRSV is divided into two genotypes, PRRSV-1 (*Betaarterivirus suid* 1, European type; prototype—Lelystad) and PRRSV-2 (*Betaarterivirus suid* 2, North American type; prototype—VR2332), which share approximately 60% genomic identity [8, 9]. Among the viral protein-coding genes, ORF5 has the highest genetic diversity and thus, has been used for lineage classification by phylogenetic analysis [10]. PRRSV-1 is classified into subtypes 1–4 (sub1–sub4) based on the ORF5 lineage classification system, while PRRSV-2 is divided into lineages 1–9 (L1–L9) [11, 12].

In South Korea, PRRSV-1 was first detected in 2005, and most of the recently isolated PRRSV-1 strains were in subgroup A in subtype 1 (sub1A) [7, 13]. In 2014, two PRRSV-1 modified live vaccines (MLVs; Unistrain PRRS (Amervac) and Porcilis PRRS) were introduced in Korea, and vaccine-like isolates (subtype 1C) were detected since 2014 [7]. PRRSV-2 was first identified in the mid-1980s and has been in circulation throughout the nation for decades [14]. Afterward, the genetic diversity of PRRSV increased at a high evolutionary rate, establishing unique country-specific lineages: lineage KOR A, B, and C (LKA, LKB, and LKC), which shows distinct genetic heterogeneity from other global lineages [7, 15]; and multiple other lineages that circulated in Korea for several decades. The NADC30-like lineage 1 or lineage 1C has been introduced into the nation by pig import since 2014, establishing more complicated PRRSV multilineage dynamics in the field [15, 16]. The newly introduced lineage 1 started to prevail in the field, and most recently it has comprised the largest population, followed by the MLV-variants lineage 5 (L5; VR2332-like) and LKB [7, 16, 17]. On top of the high mutation rate of PRRSV, the virus rapidly evolves under immune pressure caused by the use of MLV vaccines in the field or through recombination or pig movement both domestically and internationally [2, 3, 18]. Given the complicated genetic and antigenic diversity, along with varied virulence, it is crucial to continuously monitor and characterize the pathogenicity of currently circulating PRRSVs [2, 19]. In the major pork-producing countries, including the United States and China, representative PRRSV-2 strains have been selected through ORF5-based genetic surveillance as well as through the identification of genomic information, and their virulence has been confirmed through animal experiments [20].

Since virulent or highly pathogenic PRRSVs, such as JXA1 [21], HEB1 [21], HUB2 [21], JX143 [22], SY0608 [23], HuN4 [24], GSWW [25], NADC30 [19], and NADC34 [26], have been characterized, the pathogenicity of newly identified PRRSV isolates has been standardized or measured through animal experiments by comparison with the aforementioned “reference” strains. Thus, scientists, field veterinarians, and pig practitioners predict the approximate pathogenicity of prevalent PRRSV strains in the field by comparing their genetic or genomic homology to those “reference” strains. Although there have been periodic investigations of PRRSV epidemiology and genetic characteristics in Korea [7, 13–17], animal experiments have been rare, and the pathogenicity of currently circulating PRRSV-2 lineages, including unique Korean lineages (LKA, LKB, and LKC), in Korea is largely unknown.

Generally, the pathogenicity of PRRSV is assessed based on clinical symptoms (body temperature, lethargy, and body weight gain), laboratory results (antigen and antibody levels), and necropsy findings (gross observations and histopathological findings) [27, 28]. However, the multitude of assessment categories makes intuitive comparisons between different strains challenging. Therefore, several analysis techniques could be utilized such as principal component analysis (PCA), which detects the primary characteristics of data while considering their correlations [29], or meta-analysis, which comprehensively synthesizes diverse research outcomes and statistically summarizes them to draw general conclusions [30, 31].

Therefore, the objective of this study was to characterize the pathogenicity of Korean PRRSV isolates, and ultimately designate “reference” strains that represent pathogenicity according to their ORF5 gene-based lineage. Additionally, we utilized various analytical methods, including meta-analysis and PCA, to integrate the results of the identified categories with similar characteristics, aiming to facilitate an intuitive comparison of distinct strains.

## 2. Materials and Methods

### 2.1. Cells and PRRSV Isolates

**Figure 1:**
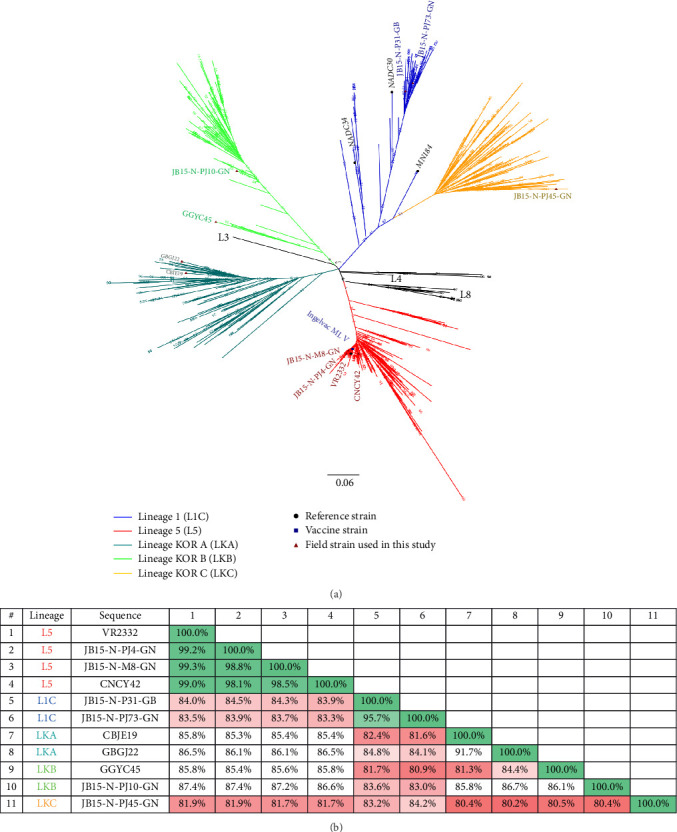
Results of genetic analysis of representative PRRSV-2 strains used in this study. (A) Phylogenetic tree analysis based on ORF5 nucleotide sequences of Korean PRRSV-2 isolates and representative reference strains. The color of the line in the phylogenetic tree indicates the lineage to which PRRSV belongs (blue: lineage 1, red: lineage 5, dark green: Korean lineage A, light green: Korean lineage B, and dark yellow: Korean lineage C). The GTRGAMMA nucleotide substitution model was used to generate the tree with 1000 bootstrap replicates by RAxML-NG. (B) ORF-wise percentage of nucleotide sequence homology between the different viruses used in this study. The lower the homology of ORF5, the redder the color; the higher the homology, the greener the color. GTRGAMMA, generalised time reversible (GTR) GAMMA; L1C, lineage 1C; L5, lineage 5; LKA, lineage KOR A; LKB, lineage KOR B; LKC, lineage KOR C; ORF, open reading frame; PRRSV, porcine reproductive and respiratory syndrome virus; RAxML-NG, randomized axelerated maximum likelihood-next generation.

Ten Korean PRRSV isolates were selected as representative or “reference” strains for each lineage circulating in Korea based on previous studies that analyzed ORF5 gene epidemiology [16] and genomic characteristics [7] by our group. The information on the isolates is summarized in Table 1. One or two strains were selected from Korean-specific lineages (LKA, LKB, and LKC) and the Korean NADC30-like clade (L1C), which are genetically distinct from the strains of other nations (Figure 1 and Figure S1). For virus isolation, PRRSV-positive samples were inoculated into MARC-145) cells, which are an African green monkey kidney epithelial cell line and highly permissive to PRRSV. When PRRSV isolation from MARC-145 cells failed, the samples were inoculated into porcine alveolar macrophages (PAMs), which are primary cells for PRRSV isolation. VR2332 (GenBank: AY150164), a prototype strain of PRRSV-2, was used for comparison with other Korean PRRSV isolates and propagated in the MARC-145 cell line. PRRSV isolates were cultured in MARC-145 cells or PAMs in Roswell Park Memorial Institute (RPMI)-1640 medium supplemented with heat-inactivated 10% fetal bovine serum (Invitrogen, Carlsbad, CA, USA) and 1% antibiotic–antimycotic solution (Invitrogen) at 37°C in a humidified 5% CO_2_ atmosphere. In addition, all of the PRRSV strains were prepared for inoculation within three passages in cells to minimize phenotypic changes.

### 2.2. Animal Study

Animal experiments were conducted in a total of four trials. The negative control (NC) group consisted of six pigs, and each infected group consisted of eight pigs. The 1st, 2nd, and 3rd animal trials included a NC group and three infection groups, and the 4th animal trial included a NC group and two infection groups (Table 2).

**Figure 2:**
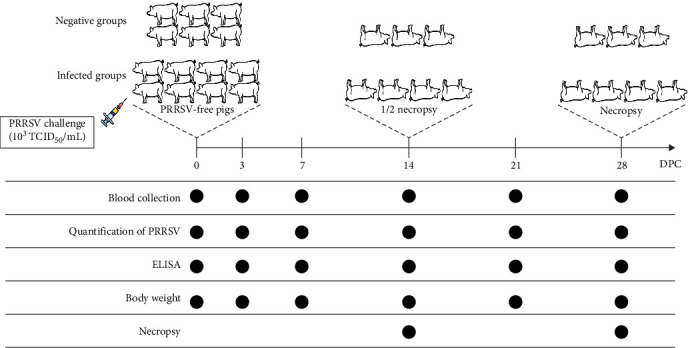
Schematic diagrams of the study design. Animal experiments were conducted in a total of four trials. For each trial, the infected and noninfected groups were populated as shown in the figure. The pigs in each infected group were intramuscularly inoculated with 2 mL of 10^3^ TCID_50_/mL of the corresponding PRRSV-2 strains for their group. Sample collection was conducted at specific time points. DPC, days postchallenge; ELISA, enzyme-linked immunosorbent assay; PRRSV, porcine reproductive and respiratory syndrome virus; TCID_50_, 50% tissue culture infective dose.

In the experiment, 4-week-old pigs were used that were purchased from a PRRSV-free farm. The pigs were randomly assigned to and housed in separate rooms after confirming their PRRSV-negative through antigen and antibody tests. The pigs in each room were numbered and provided unlimited food and water. The pigs in each infected group (*n* = 8) were intramuscularly inoculated with 2 mL of 10^3^ 50% tissue culture infective dose (TCID_50_)/mL of the corresponding PRRSV strains for their group. The pigs in the NC group (*n* = 6) remained uninfected. All pigs were tested for body temperature for 10 days after the virus challenge. Four pigs in each group were humanely euthanized 14 and 28 days postchallenge (DPC) following proper ethical guidelines. The overall animal study design is presented in Figure 2.

Serum was collected at 0 (before challenge), 3, 7, 14, 21, and 28 DPC and used to measure viral loads and the level of PRRSV-specific antibodies (immunoglobulin G (IgG)). Nasal swab samples were collected at 0, 3, 7, and 14 DPC and were used to measure the antigens released from the pigs. Pigs were also weighed at 0, 3, 7, 14, 21, and 28 DPC, from which the average daily weight gain (ADWG) was calculated. All lung tissues were collected in conical tubes on necropsy day (14 and 28 DPC) and stored immediately at −80°C until processing. The lung tissues were used to measure the viral loads and were also preserved in 10% neutral-buffered formalin for histopathological examination.

The animal experiment protocol was approved by the Jeonbuk National University Institutional Animal Care and Use Committee (JBNU 2021-095) and was performed under the guidelines and regulations detailed by the committee.

### 2.3. Quantification of the Viruses in the Samples

Viral RNA was extracted from 100 μL of serum, pretreated nasal swabs, and 1 g of lung tissue using the MagMAX Viral RNA Isolation Kit (Ambion, Applied Biosystems, Life Technologies Inc., Carlsbad, CA, USA) according to the manufacturer's instructions. The measurement of viral loads in the samples was performed by real-time quantitative reverse transcription polymerase chain reaction (qRT-PCR) using a one-step qRT-PCR kit (Prime-Q PCV2, PRRSV Detection Kit, GeNet Bio Inc., Daejeon, South Korea) according to the manufacturer's instructions with a 7500 Fast Real-Time PCR system (Applied Biosystems, Foster City, CA, USA). To measure the virus in each sample, the *Ct* values were converted to PRRSV titers (TCID_50_/mL) using standard curves generated from the titration of each PRRSV strain. To compare the different groups, the viremia and nasal swabs were converted to the area under the curve (AUC) [32].

### 2.4. Detection of Anti-PRRSV Antibodies

The sera were tested for anti-PRRSV specific antibody (IgG) against the PRRSV nucleocapsid protein using a commercially available enzyme-linked immunosorbent assay (ELISA) kit (PRRS Ab ELISA 4.0; BioNote Inc., Hwaseong-si, South Korea) according to the manufacturer's instructions. The anti-PRRSV antibody was considered positive as the sample-to-positive (S/P) ratio was ≥0.4.

### 2.5. Histopathologic Examination

Approximately 2 cm^3^ of lung tissue was fixed in 10% phosphate-buffered formalin and the tissue was routinely processed and embedded in paraffin. Tissue sections, 4 μm thick, were cut using a microtome (HM-340E; Thermo Fisher Scientific, Inc., Waltham, MA, USA), and placed on glass slides. Standard methods were used to conduct hematoxylin and eosin (H&E) staining. The microscopic lung lesion score system was modified from a previous study [33]. Briefly, the microscopic lung lesions related to interstitial pneumonia, the representative pulmonary lesion of PRRSV infection, were scored on a scale from 0 to 4 (0: no lesion, 1: mild interstitial pneumonia, 2: moderate multifocal interstitial pneumonia, 3: moderate diffuse interstitial pneumonia, and 4: severe interstitial pneumonia).

### 2.6. Data Analysis

The high fever rate and mortality rates were calculated using the following formulas:

  $$\begin{array}{c} \text{High fever rate}=\frac{\text{Number of occurences of high temperature}\left(>40.4{}^{\circ}\text{C}\right)}{\left(\text{Observation days}\times \text{number of pigs}\right)}, \end{array}$$

  $$\begin{array}{c} \text{Mortality rate}=\frac{\text{Number of death}}{\left(\text{Observation days }\times \text{number of pigs}\right)}. \end{array}$$

The Spearman correlation coefficients between all pairs of variables (viremia AUC, nasal viral load AUC, high fever rate, mortality rate, lung viral loads, ADWG, and microscopic lung lesion score) were calculated. PCA was also conducted to assess the covariation for all seven groups, and the potential clustering of genotypes based on clinical outcome. The strainwise correlations of clinical outcomes resulting from PCA were all simultaneously represented in the correlation biplot.

A meta-analysis was done to integrate the experimental results into a unified metric for evaluating the pathogenicity of the PRRSV-2 strains. To compare the results for each strain with the overall PRRSV results, a meta-analysis was conducted on seven outcomes (viremia AUC, nasal viral load AUC, lung viral loads, high fever rate, microscopic lung lesion score, ADWG, and mortality rate) from each strain at 14 DPC, measuring the standardized mean differences (SMDs; Hedges'g). The SMD is a standardized effect size making it possible to compare mean differences on the same basis even if outcomes have different units of measurement [34]. The data were pooled using the inverse variance method [31], and a random effects model was used to measure the SMD with 95% confidence intervals (CIs), considering the variation among the 11 challenged strains. The Knapp–Hartung adjustment was used to account for the uncertainty of the estimates due to the heterogeneity of the PRRSV strains [30]. In this way, the meta-analysis enabled us to assess the variations among PRRSV strains equally and summarize the results across PRRSV strains. The seven outcomes were categorized into two indicators: “excretion” (viremia AUC, nasal viral load AUC, and lung viral loads) and “clinical signs” (high fever rate, microscopic lung lesion score, ADWG, and mortality rate). To quantify the excretion and clinical signs for each strain, a network meta-analysis of the excretion indicators and clinical sign indicators was performed using the SMD and standard error (SE) calculated in the above meta-analysis. Unlike other variables, ADWG was integrated with negative values, as lower values indicate more severe signs. Network meta-analysis, which allows for the comparison and analysis of intervention effects based on both direct and indirect evidence, aids in indirectly comparing pathogenicity differences among strains when a direct comparison is not feasible [35, 36]. Exploiting these findings in the network meta-analysis, we grouped similar correlated outcomes into one indicator. The network meta-analysis results comparing each of the 11 PRRSV strains around the overall PRRSV outcomes were presented as forest plots with SMDs and 95% CIs.

In summary, we compared the mean overall incidence of PRRSV and that of each individual strain by calculating their SMDs and SEs. Using network meta-analysis, we integrated the SMDs and SEs for each indicator to compare pathogenicity differences between strains. With this methodology, we intended to integrate the direct comparisons between PRRSV strains and compare the outcomes of every strain simultaneously.

GraphPad Prism 9.3.1 software (San Diego, CA, USA) was used to generate graphs, and Statistical Package for the Social Sciences (SPSS) Advanced Statistics 17.0 software (Chicago, IL, USA) was used for statistical analysis. The significance of differences in ADWG, viral loads, ELISA, and microscopic lung lesion scores was analyzed through one-way analysis of variance (ANOVA) with Tukey's multiple comparisons test. The correlation matrix was visualized using the “psych” package [37] in R version 4.1.1 [38] (R Core Team, Vienna, Austria). PCA was performed using the prcomp function in R software [38]. The meta-analysis was conducted with the “meta” and “netmeta” packages in R software [38].

## 3. Results

### 3.1. Clinical Symptoms

#### 3.1.1. Mortality

As shown in Table 3, one pig died in the groups infected with JB15-N-PJ73-GN (L1C), GBGJ22 (LKA), and JB15-N-PJ45-GN (LKC) on 24, 10, and 13 DPC, respectively (mortality rate: 12.5%). The dead pigs of these strain-infected groups commonly showed bronchopneumonia or pleuropneumonia in their lung tissue. In the GGYC45 (LKB) infected group, two pigs died at 10 and 17 DPC. The pig that died at 10 DPC had a fever of 41.2°C and weighed only 7 kg at the time. The pig that died at 17 DPC had observed edema, congestion, and pleuropneumonia in the lung tissue. In the JB15-N-PJ10-GN (LKB) infected group, one pig died at 10 DPC and two pigs died at 11 DPC. The remaining pigs in the JB15-N-PJ10-GN (LKB) infected group were humanely euthanized as per Institutional animal care and use committee (IACUC) guidelines at 14 DPC because they showed severe respiratory symptoms such as coughing and tachypnea and fell into lethargy. The pigs infected with JB15-N-PJ10-GN (LKB) showed severe bronchopneumonia, pleuritis, and congestion lesions in the lung tissue. Overall, the pigs in the group infected with the LKB strains had the most severe clinical symptoms and the highest mortality rate.

#### 3.1.2. Body Temperature

**Figure 3:**
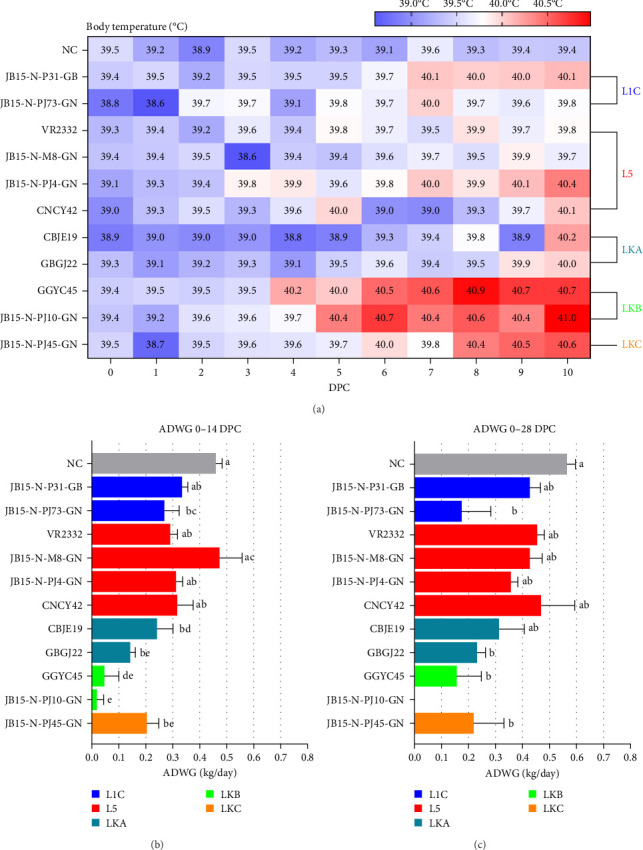
Results of clinical symptoms due to PRRSV-2 infection. (A) Changes in the body temperature of pigs caused by PRRSV-2 infection. The lower the body temperature, the bluer the color; the higher the temperature, the redder the color. Growth performance of the noninfected and infected groups. The ADWG was calculated for 0–14 DPC (B) and 0–28 DPC (C). The color of the bar in the graph indicates the lineage to which PRRSV belongs (blue: L1C, red: L5, dark green: LKA, light green: LKB, and dark yellow: LKC). The data are presented as group means ± SEM. Significant differences among the experimental groups are denoted by different letters (*p* < 0.05, Tukey's test, and two-way ANOVA). ADWG, average daily weight gain; ANOVA, analysis of variance; DPC, days postchallenge; L1C, lineage 1C; L5, lineage 5; LKA, lineage KOR A; LKB, lineage KOR B; LKC, lineage KOR C; NC, negative control; PRRSV, porcine reproductive and respiratory syndrome virus; SEM, standard error of the mean.

The pigs in the NC group had no high fever during the experiment. The rectal temperature of PRRSV-infected pigs surged as early as 4 DPC and as late as 9 DPC. Most pigs infected with PRRSV had a mean peak rectal temperature of approximately 39.7−40°C. In contrast, pigs infected with LKB strains (GGYC45 and JB15-N-PJ10-GN) exhibited a persistent fever (>40°C) from 5 to 10 DPC. LKB strain-infected groups had mean peak rectal temperatures of 40.9 and 41.0°C on 8 DPC and 10 DPC, respectively (Figure 3A). The JB15-N-P31-GB (L1C), JB15-N-PJ4-GN (L5), and JB15-N-PJ45-GN (LKC) infected groups also exhibited persistent fever (around 40°C) from 7 DPC.

#### 3.1.3. Weight Loss

The ADWG in the PRRSV-2 infected groups were significantly lower (*p* < 0.05) than that in the NC group, except for that in the JB15-N-P31-GB (L1C) and L5 strain-infected groups at 14 DPC. Compared with those in the group infected with VR2332 (prototype of PRRSV-2, L5, a well-studied strain), the L1C and L5 strain (except for JB15-N-M8-GN)-infected groups showed similar ADWG, whereas the Korean lineage strain-infected groups displayed lower ADWG. Although the LKA and LKC strain-infected groups exhibited no significant difference from the VR2332 (L5) infected group, the LKB strain-infected groups were significantly lower (*p* < 0.01) than that of the VR2332 (L5) infected group at 14 DPC (Figure 3B).

At 28 DPC, the ADWG of the JB15-N-P31-GB (L1C), L5 strain, and CBJE19 (LKA) infected groups were similar to each other and not significantly different from that of the NC group, but the ADWG of the other infected groups significantly differed (*p* < 0.05) from that of the NC group. The groups infected with JB15-N-PJ73-GN (L1C), GBGJ22 (LKA), LKB, or LKC had lower ADWG than did the VR2332 infected group, although these differences were not significant (Figure 3C). Overall, the Korean lineage strains (CBJE19, GBGJ22, GGYC45, JB15-N-PJ10-GN, and JB15-N-PJ45-GN) induced lower ADWGs up to 28 DPC than the other PRRSV-2 strains. In particular, a significant reduction in the pig growth rate was observed for the LKB strains (GGYC45 and JB15-N-PJ10-GN).

### 3.2. Quantification of the Viral Loads in Pig Samples

#### 3.2.1. Viremia

**Figure 4:**
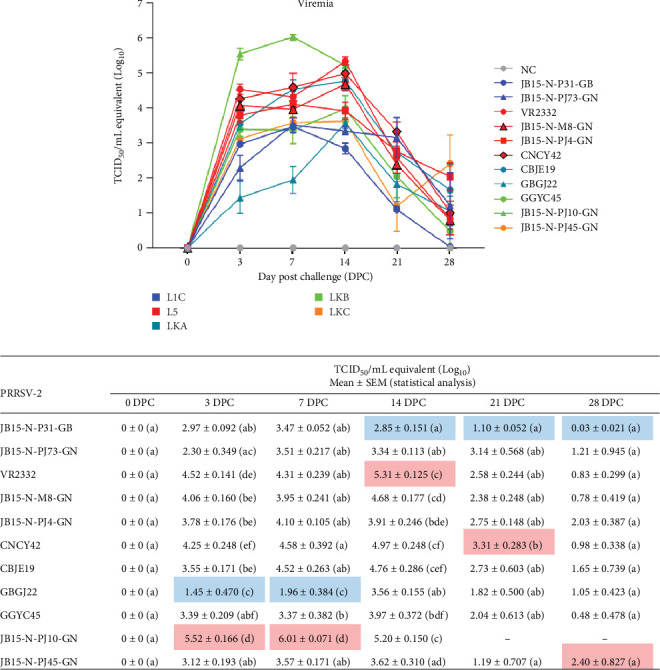
The viral loads in sera obtained from inoculated pigs. The viral loads in the serum at 0, 3, 7, 14, 21 and 28 DPC were quantified by real-time reverse transcription-PCR (qRT-PCR). The raw viremia data for each group at each time point are shown with statistical differences at the bottom. The viral titers were calculated using the standard curve of the threshold number of cycles plotted against each of the known viral titers. The color of the line in the graph indicates the lineage to which PRRSV belongs (blue: L1C, red: L5, dark green: LKA, light green: LKB, and dark yellow: LKC). The red and blue squares in the raw data table indicate the highest and lowest viral loads, respectively, at each time point. The data are presented as group means ± SEM. Significant differences among the experimental groups are denoted by different letters (*p* < 0.05, Tukey's test, and two-way ANOVA). ANOVA, analysis of variance; DPC, days postchallenge; L1C, lineage 1C; L5, lineage 5; LKA, lineage KOR A; LKB, lineage KOR B; LKC, lineage KOR C; NC, negative control; PRRSV, porcine reproductive and respiratory syndrome virus; qRT-PCR, quantitative reverse transcription polymerase chain reaction; SEM, standard error of the mean.

No viral load was detected in any of the samples in the NC group. All the PRRSV-infected groups exhibited the highest level of viral replication within 14 DPC, and the viremia decreased after 14 DPC. The JB15-N-PJ10-GN (LKB) infected group showed a marked increase (*p* < 0.05) in viremia at 3 DPC (early in the infection) compared to the other PRRSV-2 infected groups. This group peaked at 7 DPC and had significantly higher (*p* < 0.05) viremia than the other infected groups. Although the viremia of this infected group decreased slightly by 14 DPC, it was similar to that of the VR2332 (L5) infected group which showed peak viremia at 14 DPC (Figure 4).

Overall, the L5 strain, CBGJ22 (LKA), and JB15-N-PJ10-GN (LKB) infected groups exhibited moderate or greater virus replication. In particular, given its rapid increase and high viremia, the JB15-N-PJ10-GN (LKB) strain exhibited the greatest capacity for viral replication.

#### 3.2.2. Viral Loads in Nasal Swabs

**Figure 5:**
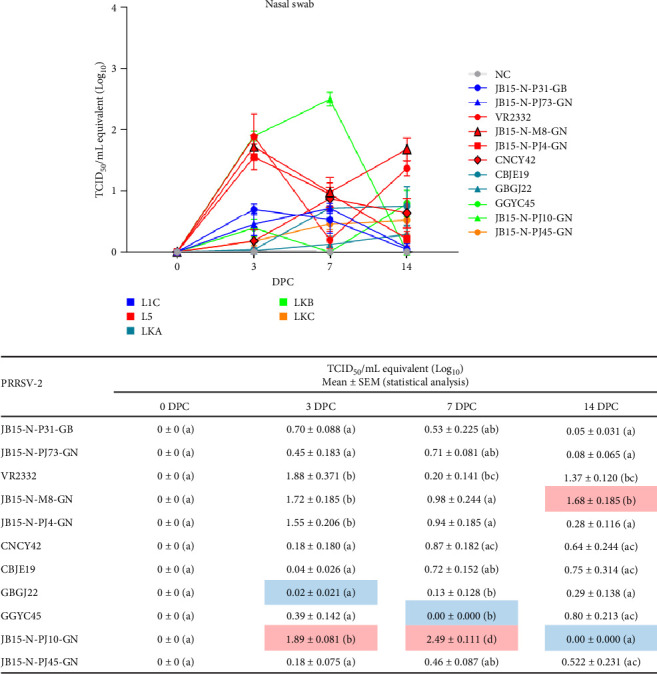
Viral loads in nasal swab samples obtained from inoculated pigs. (A) The nasal viral loads at 0, 3, 7 and 14 DPC were quantified by qRT-PCR. The raw data from each group at each time point are shown with statistical differences at the bottom. The viral titers were calculated using the standard curve of the threshold number of cycles plotted against each of the known viral titers. The color of the line in the graph indicates to which lineage PRRSV belongs (blue: L1C, red: L5, dark green: LKA, light green: LKB, and dark yellow: LKC). The red and blue squares in the raw data table indicate the highest and lowest viral loads at each time point. The data are presented as group means ± SEM. Significant differences among the experimental groups are denoted by different letters (*p* < 0.05, Tukey's test, two-way ANOVA). ANOVA, analysis of variance; DPC, days postchallenge; L1C, lineage 1C; L5, lineage 5; LKA, lineage KOR A; LKB, lineage KOR B; LKC, lineage KOR C; NC, negative control; PRRSV, porcine reproductive and respiratory syndrome virus; qRT-PCR, quantitative reverse transcription polymerase chain reaction; SEM, standard error of the mean.

No viral RNA was detected in any of the samples in the NC group. Three L5 strains (VR2332, JB15-N-M8-GN, and JB15-N-PJ4-GN) and JB15-N-PJ10-GN (LKB) infected groups showed a sharp increase in virus shedding titers at 3 DPC. The viral shedding titer of the JB15-N-PJ10-GN infection group peaked (2.49 ± 0.111) at 7 DPC, which was clearly different from that of the other infection groups (Figure 5).

Nasal swabs are the best indicator of PRRSV shedding titer, but may be subject to some error depending on the timing of sampling and the condition of the pig. Nevertheless, the JB15-N-PJ10-GN (LKB) strain had high viral shedding titers and a high capacity for viral replication.

#### 3.2.3. Viral Loads in Lung Tissues

**Figure 6:**
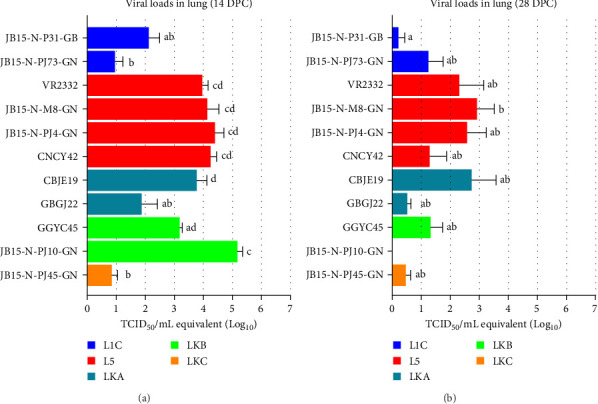
Viral loads in the lung samples obtained from pig experiments. (A) and (B) The lung viral loads at 14 DPC and 28 DPC, respectively. PRRSV RNA was detected in the lung samples using qRT-PCR. The viral titers of (A)–(B) were calculated from the standard curve of the threshold cycle number plotted against each known virus titer. The color of the bar in the graph indicates the lineage to which PRRSV belongs (blue: L1C, red: L5, dark green: LKA, light green: LKB, and dark yellow: LKC). The data are presented as group means ± SEM. Significant differences among the experimental groups are denoted by different letters (*p* < 0.05, Tukey's test, two-way ANOVA). ANOVA, analysis of variance; DPC, days postchallenge; L1C, lineage 1C; L5, lineage 5; LKA, lineage KOR A; LKB, lineage KOR B; LKC, lineage KOR C; PRRSV, porcine reproductive and respiratory syndrome virus; qRT-PCR, quantitative reverse transcription polymerase chain reaction; SEM, standard error of the mean.

No viral load was detected in any samples in the NC group at 14 or 28 DPC. At 14 DPC, similar to the results of the serum and nasal swab viral loads, the lung tissue samples from the JB15-N-PJ10-GN (LKB), CBGJ22 (LKA), and L5 strain infected groups had higher viral loads. The JB15-N-PJ10-GN (LKB) infected group had the highest mean viral load, with a value of 10^5.17^ TCID_50_ (equivalent)/mL (*p* < 0.05), which was significantly greater than that of the L1C, LKA, and LKC strain infected groups. Among the groups infected with L5 strains, CBGJ22 (LKA) and GGYC45 (LKB) exhibited moderate viral loads of approximately 10^3^–10^4^ TCID_50_/mL. The mean lung viral loads of the other remaining PRRSV-2 infected groups were approximately 10^2^ or less TCID_50_ (equivalent)/mL, which was significantly lower (*p* < 0.001) than that of the VR2332 group (Figure 6A).

At 28 DPC, an overall reduction in lung viral load was observed in all the PRRSV-2 infection groups compared to the results at 14 DPC. L5 strain (except for JB15-N-PJ4-GN) and CBJE19 (LKA) infected groups showed a mean viral load in the lung greater than 10^2.0^ TCID_50_ (equivalent)/mL. The other groups had viral loads less than this threshold (Figure 6B).

### 3.3. Serology

**Figure 7:**
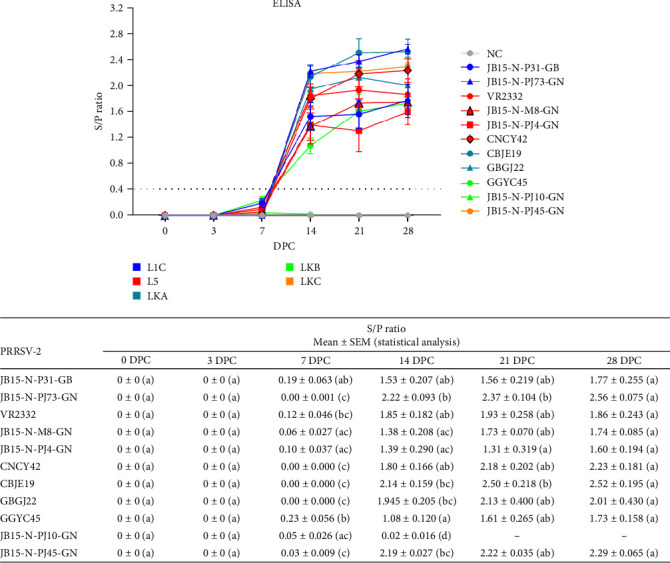
The kinetics of the PRRSV-specific IgG antibody response against PRRSV-2 infection in pigs. The PRRSV-specific antibody titer in the pigs was measured by ELISA. The dashed line indicates the designated threshold value (S/P ratio of 0.4). The raw data from each group at each time point are shown with statistical differences at the bottom. The color of the line in the graph indicates the lineage to which PRRSV belongs (blue: L1C, red: L5, dark green: LKA, light green: LKB, and dark yellow: LKC). The data are presented as group means ± SEM. Significant differences among the experimental groups are denoted by different letters (*p* < 0.05, Tukey's test, two-way ANOVA). ANOVA, analysis of variance; DPC, days postchallenge; ELISA, enzyme-linked immunosorbent assay; IgG, immunoglobulin G; L1C, lineage 1C; L5, lineage 5; LKA, lineage KOR A; LKB, lineage KOR B; LKC, lineage KOR C; NC, negative control; PRRSV, porcine reproductive and respiratory syndrome virus; S/P, sample-to-positive; SEM, standard error of the mean.

The response of PRRSV-specific antibodies (IgG) in pigs after PRRSV-2 infection was measured by an ELISA based on the nucleocapsid (N) protein. The pigs in the NC group remained seronegative throughout the experiment period, while all the pigs in the PRRSV-2-infected groups were seropositive at 14 DPC, which continued until 28 DPC (Figure 7). In most of the PRRSV-infected groups, the S/P ratio increased rapidly between days 7 and 14, peaked on day 21, and tended to hold steady with similar S/P ratios until 28 DPC. In comparison to the other PRRSV-2 infection groups, the JB15-N-PJ10-GN (LKB) infection group, in which pig mortality was high, had a significantly lower level of S/P ratio. High viral loads did not always result in high S/P ratios, and antibody titer levels were strain-dependent.

### 3.4. Histopathology

**Figure 8:**
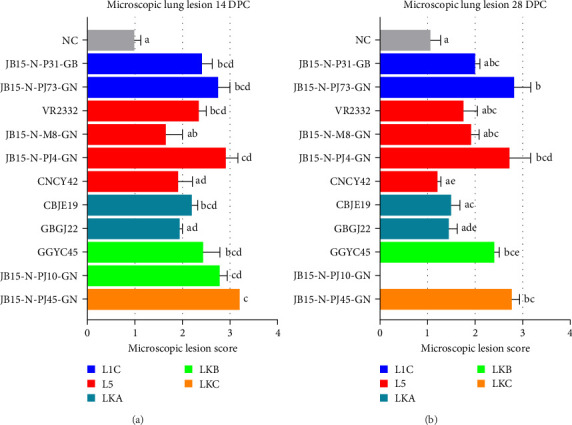
Evaluation of microscopic lung lesions in the pigs on each necropsy day. (A) 14 DPC. (B) 28 DPC. H&E stained lung sections were used for scoring microscopic lesions in pigs on a 5-point scale (0: no lesion, 1: mild interstitial pneumonia, 2: moderate multifocal interstitial pneumonia, 3: moderate diffuse interstitial pneumonia, and 4: severe interstitial pneumonia). The color of the bar in the graph indicates the lineage to which PRRSV belongs (blue: L1C, red: L5, dark green: LKA, light green: LKB, and dark yellow: LKC). The data are presented as group means ± SEM. Significant differences among the experimental groups are denoted by different letters (*p* < 0.05, Tukey's test, two-way ANOVA). H&E, hematoxylin and eosin; ANOVA, analysis of variance; DPC, days postchallenge; L1C, lineage 1C; L5, lineage 5; LKA, lineage KOR A; LKB, lineage KOR B; LKC, lineage KOR C; NC, negative control; PRRSV, porcine reproductive and respiratory syndrome virus; SEM, standard error of the mean.

At 14 DPC, all of the groups exhibited significantly higher (*p* < 0.05) microscopic lung lesion scores than the NC group, but JB15-N-M8-GN (L5), CNCY42 (L5), and GBGJ22 (LKA) infected groups had no significant difference. The JB15-N-PJ45-GN (LKC) infected group had the highest mean microscopic lung lesion score (3.2). Following the JB15-N-PJ45-GN (LKC) strain were the L1C, VR2332 (L5), JB15-N-PJ4-GN (L5), and LKB strains, which induced similar lung lesion scores (Figure 8A). In the case of the JB15-N-PJ10-GN (LKB)-infected group, although all the pigs in this group died up to 11 DPC, they had the second highest microscopic lung lesion score. There was no significant difference in the microscopic lung lesion score compared to that of the VR2332 (prototype of PRRSV-2) infected group across all the infected groups. By 28 DPC, the mean microscopic lesion scores had decreased in most infected groups, but the scores of the JB15-N-PJ73-GN (L1C), JB15-N-PJ4-GN (L5), GGYC45 (LKB), and JB15-N-PJ45-GN (LKC) groups were comparable to those of the 14 DPC group (Figure 8B). There was no significant difference in the microscopic lung lesion score compared to that of the VR2332 in any infected group.

### 3.5. Correlation Analysis and PCA

**Figure 9:**
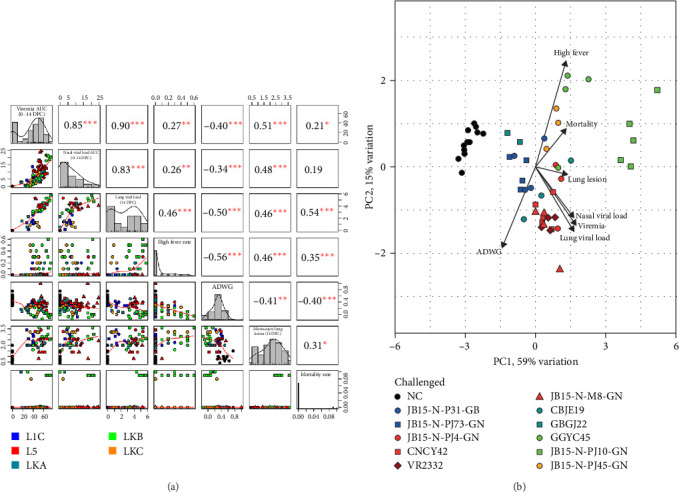
Results of correlation and PCA between the data obtained from the animal experiments. (A) The correlations between the seven variables (viremia AUC, nasal viral load AUC, high fever rate, mortality rate, lung viral loads, ADWG, and microscopic lung lesion score) were evaluated and visualized using the “psych” package in R version 4.1.1. (B) The seven variables were input into multivariate analysis (PCA). PCA was conducted using the prcomp function in R software. The color of the mark in the graph indicates the lineage to which PRRSV belongs (blue: L1C, red: L5, dark green: LKA, light green: LKB, and dark yellow: LKC). Statistically significant differences are indicated at *p* < 0.05(*⁣*^*∗*^),  *p* < 0.01(*⁣*^*∗∗*^),  *p* < 0.001(*⁣*^*∗∗∗*^). ADWG, average daily weight gain; AUC, area under the curve; DPC, days postchallenge; L1C, lineage 1C; L5, lineage 5; LKA, lineage KOR A; LKB, lineage KOR B; LKC, lineage KOR C; NC, negative control; PCA, principal component analysis; PRRSV, porcine reproductive and respiratory syndrome virus.

Correlation analysis was performed on data from all the PRRSV-2 infected groups up to 14 DPC, since the peak viral load was detected within 14 DPC, indicating that the results at 14 DPC may reflect the maximal influence of PRRSV infection on the host. Overall, all variables showed statistically significant negative or positive correlations except for the correlation between nasal viral load and mortality rate (Figure 9A). The results associated with virus replication, such as the viral loads in serum, nasal swabs and lung tissues showed the highest positive correlation (*R* > 0.8 and *p* < 0.001). The ADWG showed a significant negative correlation (*p* < 0.01) with all the other variables. Furthermore, a high fever rate, histopathological lung lesion score, and mortality rate, which are related to clinical signs of PRRSV infection, were positively correlations with each other and with variables related to virus replication.

PCA provided a satisfactory ordination of the correlation composition across the strains, the eigenvalues of the first two components accounting for 74% (59% and 15% for principal component 1 (PC1) and principal component 2 (PC2), respectively) of the total variance of clinical outcomes (Figure 9B). JB15-N-PJ10-GN (LKB) was positively correlated with mortality and lung lesions, whereas GGYC45 (LKB) was positively correlated with a high fever rate. L5 strains, including the reference strain VR2332 (L5) and other MLV variants (L5), were positively correlated with variables related to virus replication.

### 3.6. Meta-Analysis

**Figure 10:**
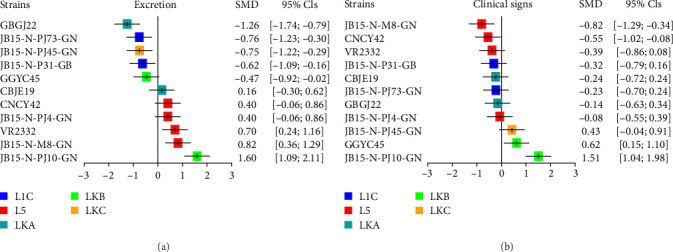
Results of a meta-analysis of seven variables obtained from pig experiments. (A) Three variables (viremia AUC, nasal viral load AUC, and lung viral loads) made up the “excretion” indicator. (B) Four variables (high fever rate, microscopic lung lesion score, ADWG, and mortality rate) made up the “clinical signs” indicator. The data from the overall PRRSV-2 infected group and the data from individual infected groups were compared, and the SMDs were calculated for each strain-specific variable. The meta-analysis was performed with the “meta” and “netmeta” packages in R software. The results were investigated using a random effect model with 95% CIs to account for the variation among the eleven challenged PRRSV-2 strains. The color of the square in the graph indicates the lineage to which PRRSV belongs (blue: L1C, red: L5, dark green: LKA, light green: LKB, and dark yellow: LKC). ADWG, average daily weight gain; AUC, area under the curve; CIs, confidence intervals; L1C, lineage 1C; L5, lineage 5; LKA, lineage KOR A; LKB, lineage KOR B; LKC, lineage KOR C; PRRSV, porcine reproductive and respiratory syndrome virus; SMDs, standardized mean differences.

Based on the results of the correlation analysis, we integrated the viral loads in serum, nasal swabs, and lung tissue using network meta-analysis to create an “excretion” indicator. We also combined the results of high fever rates, high mortality rates, ADWG, and microscopic lung lesion scores to form the “clinical signs” indicator. According to the excretion data, the JB15-N-PJ10-GN (LKB) infected group showed the most excretion among the PRRSV-2 infected groups, followed by the L5 strain and CBJE19 (LKA) infected groups. These infected groups did not significantly differ from the VR2332 infected group, whereas the GGYC45 (LKB), L1C strain, JB15-N-PJ45-GN (LKC), and GBGJ22 (LKA) infected groups displayed significantly lower (*p* < 0.05) excretion than the VR2332 infected group (Figure 10A). Overall, the JB15-N-PJ10-GN (LKB) and L5 strain infected groups ranked higher than other infected groups, indicating that the JB15-N-PJ10-GN (LKB) and L5 strains were better able to transmit themselves from pig to pig.

In terms of clinical signs, the JB15-N-PJ10-GN (LKB) infected group exhibited the most clinical signs among the PRRSV-infected groups, followed by the GGYC45 (LKB), JB-15-N-PJ45-GN (LKC), and JB-15-N-PJ4-GN (L5) infected groups. The LKB strain infected groups had a statistical significance of the difference in clinical signs with the VR2332 infected group (Figure 10B). The LKB and LKC strains were ranked highly, whereas the other PRRSV-2 strains showed similar clinical signs. The JB15-N-M8-GN strain exhibited the lowest clinical signs among the PRRSV-2 strains used in this study. Overall, the JB15-N-PJ10-GN infected group had higher excretion and more clinical signs than the other PRRSV-infected groups.

## 4. Discussion

One of the characteristics of PRRS is the wide range of clinical signs, which could include asymptomatic animals infected with a low-virulence PRRSV strain and severe disease caused by a virulent PRRSV strain [20, 39]. Virulent PRRSV infection is characterized by high mortality, high fever (40.5–42.0°C), high viral loads, and secondary infections with other bacteria [20]. Although the widespread clinical manifestations of PRRS have given rise to the terms “virulent” and “highly virulent” (or “highly pathogenic”), there are currently no precise criteria for classifying PRRSV pathogenicity. In general, the pathogenicity of novel PRRSVs was demonstrated through comparative animal experiments with representative reference PRRSV strains, which are well-studied. However, the circulation of different PRRSV-2 lineages shows a strong geographic association (Figure S1) [12]. In Korea, not only globally prevalent MLV-variants of L5, but also country-specific lineages (LKA, LKB, and LKC) are circulating in the field. Additionally, within globally emerging L1 viruses, which is now the most prevalent lineage in Korea, the sublineage L1C of different nations (United States, China, and Korea) shows distinct genetic differences even though they are in the same lineage (Figure S1). Therefore, if we want to standardize the assessment of the pathogenicity of viruses from a specific region, the results of viruses isolated from that specific region should be considered, as genetically distinct region-specific lineages could show distinct antigenic characteristics.

**Figure 11:**
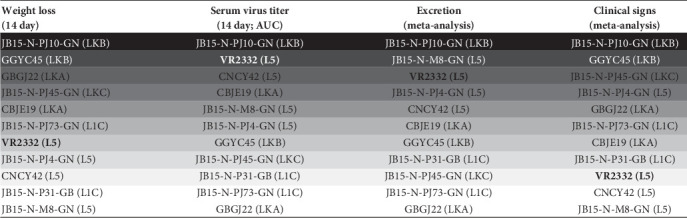
Summary of clinical findings and indicators in order of decreasing severity. Shaded boxes indicate the most (black) to the least (white) isolates for the respective indicators. AUC, area under the curve; L1C, lineage 1C; L5, lineage 5; LKA, lineage KOR A; LKB, lineage KOR B; LKC, lineage KOR C.

In this study, 10 representative PRRSV-2 isolates, selected from five lineages (L1C, L5, LKA, LKB, and LKC) of PRRSV-2 prevalent in Korea [7, 16], as well as the reference strain, VR2332, were investigated for their pathogenicity through four animal experiments on 4-week-old weaned piglets. Although the PRRSV strains used in this study were isolated in the years between 2011 and 2015, the strains were selected based on previously described genomic characteristics [7] and as the parental strains of currently circulating viruses in Korea. We performed necropsy on half of the experimental groups at 14 DPC, the time when PRRSV is likely to have the highest viral load within the host [40–43], and on the other half of the experimental group at 28 DPC. In addition, the body temperature and mortality which were difficult to compare intuitively were calculated as rates to facilitate comparison. The data of pigs that died before necropsy day (14 DPC and 28 DPC) were subordinated to the 14 DPC or 28 DPC results. To overcome and minimize batchwise effects of separate animal experiments, we integrated the 14 DPC data and summarized them by meta-analysis, which extends the sample size and statistical power to further improve the effect size estimate [44]. Based on the correlation analysis and empirical evidence, the seven outcomes of interest (viremia AUC, nasal viral load AUC, high fever rate, mortality rate, lung viral loads, ADWG, and microscopic lung lesion score) were classified as “excretion” (viral loads within serum and nasal swab and lung) or “clinical signs” (mortality, weight gain, fever, and lung lesion). Hence, comparisons and direct evidence of outcomes between PRRSV strains were quantitatively integrated for each corresponding classified indicator through a frequentist network meta-analysis. Because frequentist network meta-analysis computes !probabilities under the assumption that given data are repeated indefinitely, the results are presented as point estimates with 95% CIs, which are traditional and easy to interpret [45]. As a result, it is simple to understand and summarize comparisons of effects between PRRSV-2 strains in this experiment, better than the classical way to evaluate PRRSV virulence mainly by weight loss and serum viremia (Figure 11), offering insightful information.

The strain VR2332, a prototype of PRRSV-2 and the parent strain of the MLV, is known to induce moderate lung lesions [46, 47]. The VR2332-based L5 MLV, Ingelvac PRRS MLV, exhibits attenuated virulence and has been used not only globally but also domestically in Korea for decades since its commercialization in 1996 in Korea [15, 48]. Despite their long usage, MLVs generally have known safety issues, such as reversion to virulence and persistence and circulation in the field of vaccine-derived strains [48]. In this study, the pathogenicity of the Korean L5 MLV-variant strains JB15-N-M8-GN, JB15-N-PJ4-GN, and CNCY42, which showed relatively high genetic homology with the VR2332-based MLV vaccine (99.3%, 99.2%, and 98.7% in ORF5 level, respectively) was evaluated. According to our meta-analysis, these strains exhibited high levels of excretion compared to the other PRRSV-2 isolates used in this study but showed low levels of clinical signs, except for JB15-N-PJ4-GN, which displayed the 4th most clinical signs (Figures 9B and 10). As the Korean swine industry has applied the nationwide L5 MLV for decades and the L5 MLV variants have been predominantly circulating in the field [15, 16], these results indicate that the attenuated MLV could regain its higher excretion ability with or without higher pathogenicity, which would be enough to persist and circulate in the field and would make it more likely to mutate and recombine with field strains [7, 16].

Among the Korean-specific lineages (LKA, LKB, and LKC) which are genetically distinct from the viruses of other nations (Figure S1), the strain JB15-N-PJ10-GN (LKB) was investigated as the most virulent strain by both classical virulence indicators (weight loss and serum virus titer) and indicators of meta-analysis (Figures 10 and 11). Additionally, another strain of the LKB lineage, GGYC45, showed the second-highest weight loss and second-highest rank in clinical sign indicator, but it did not show either high serum viremia or a high rank as a excretion indicator compared to the other isolates (Figure 11). Although the JB15-N-PJ10-GN and GGYC45 strains are classified in the LKB family, they showed relatively high sequence distances within the ORF5 gene (13.9%), putting them into distinct subclades (Figure 1A). Additionally, the GGYC45 strain has been investigated as a recombinant strain with major parent LKC and minor parent Ingelvac MLV [7]. As the LKB strains continue to circulate in the field and most recent isolates are genetically close to JB15-N-PJ10-GN (Figures 1A and S1) [16], it is highly plausible that the high excretion and virulence of this highly pathogenic Korean lineage have led the viruses to evade the immunological barrier induced by the MLV vaccine in herds. Continuous surveillance and further experiments regarding the cross-protective efficacy of currently commercialized vaccines against highly virulent LKB viruses should be conducted. On the other hand, the LKA and LKC viruses showed lower levels of excretion than the JB15-N-PJ10-GN strain and L5 viruses, but higher clinical signs than the low-virulence L5 MLV variants (JB15-N-M8-GN and CNCY42) and VR2332 (Figures 10 and 11). These results might explain why these moderately pathogenic lineages (LKA and LKC) were less prevalent in the field than L5 MLV variants and LKB, which are more transmissible [16].

The lineage 1C (NADC30-like) viruses tested in this study (JB15-N-P31-GB and JB15-N-PJ73-GN) showed low or moderate levels of excretion and clinical signs (Figures 10 and 11). According to the first report of the NADC30 strain in the US in 2008, the virus showed high virulence with severe respiratory symptoms [19], and these NADC30-like or L1C viruses have been predominant from 2009 to 2014 until the emergence of NADC34-like or L1A viruses in the United States since 2014 [18]. Subsequently, variant NADC30-like started to infect Chinese pigs in 2014 and became prevalent in China [49]. In Chinese cases, genetically variant NADC30-like viruses showed highly variable clinical symptoms, ranging from inapparent to severe symptoms, and these recombination characteristics are thought to contribute to the various pathogenicity [49, 50]. In Korea, L1C or NADC30-like viruses have been imported since 2014, and they have become the most prevalent lineage in the field [16, 17]. Thus, although the Korean NADC30-like viruses tested in this study showed relatively low pathogenicity, these viruses might not be able to recapitulate the pathogenicity of the currently circulating L1C PRRSV-2 in Korea because the tested strains were isolated in 2015. Indeed, Korean NADC30-like viruses have continuously evolved and shown a level of genetic diversity in the ORF5 gene ranging from 86.7% to 100% (Figure 1A); moreover, recent identification of recombinant L1C viruses in Korea indicates that the genomic diversity of NADC30-like viruses in Korea is also increasing [7, 51]. As other lineage 1 viruses, including L1A and L1B, have been recently imported into Korea and because their genetic diversity is expanding [16, 17, 52], further investigation is needed.

## 5. Conclusions

To the best of our knowledge, this is the first study to systematically investigate the virulence of genetically divergent Korean PRRSV-2 through pig experiments and to attempt to define the level of lineage-wide pathogenicity. Various analytical tools, including meta-analysis, were utilized in our study to summarize and evaluate clinical outcome variables. Among the multiple lineages circulating in Korea, LKB is the most highly pathogenic lineage showing the highest excretion levels and clinical signs along with the representative strain JB15-N-PJ10-GN. L5 MLV-variant strains showed high levels of excretion but mild clinical signs, explaining the consistently high prevalence of these viruses in the field. Other Korean lineages (LKA and LKC) and recently emerging L1C viruses showed lower levels of excretion than LKB and L5 viruses but also moderate levels of clinical signs, between those of LKB and L5. Given that PRRSV-2 continually mutates and changes its dominant lineage, continuous surveillance and pathogenicity assessment should be conducted on newly emerging PRRSV-2 strains. This study offers useful insights into the multiple lineage dynamics of PRRSV-2.

## Figures and Tables

**Table 1:** Information on the PRRSV isolates used in this study.

Strain	Isolated	Propagated in	ORF5 lineage	Accession no.
JB15-N-P31-GB	2015	PAM	L1 (NADC30-like)	MZ287320
JB15-N-PJ73-GN	2015	PAM	L1 (NADC30-like)	MZ287317
JB15-N-M8-GN	2015	MARC-145	L5 (VR2332-like)	MZ287322
JB15-N-PJ4-GN	2015	PAM	L5 (VR2332-like)	MZ287323
CNCY42	2011	MARC-145	L5 (VR2332-like)	MZ287319
CBJE19	2010	PAM	LKA (Nation-specific)	MZ287316
GBGJ22	2011	PAM	LKA (Nation-specific)	MZ287315
GGYC45	2010	PAM	LKB (Nation-specific)	MZ287324
JB15-N-PJ10-GN	2015	PAM	LKB (Nation-specific)	MZ287321
JB15-N-PJ45-GN	2015	MARC-145	LKC (Nation-specific)	MZ287318

Abbreviations: L1, lineage 1; L5, lineage 5; LKA, lineage KOR A; LKB, lineage KOR B; LKC, lineage KOR C; MARC-145, African green monkey kidney epithelial cell line; ORF5, open reading frame 5; PAM, porcine alveolar macrophage; PRRSV, porcine reproductive and respiratory syndrome virus.

**Table 2:** Summary of the design of the four animal experiments.

Animal experiment	Noninfected group (number of pigs)	PRRSV-2 infected groups (number of pigs per group)	Inoculated PRRSV-2 strains
1st animal trial	1 (*n* = 6)	3 (*n* = 8)	VR2332, JB15-N-M8-GN, GGYC45
2nd animal trial	1 (*n* = 6)	3 (*n* = 8)	JB15-N-PJ10-GN, JB15-N-PJ45-GN, JB15-N-PJ73-GN
3rd animal trial	1 (*n* = 6)	3 (*n* = 8)	JB15-N-PJ4-GN, JB15-N-P31-GB, CNCY42
4th animal trial	1 (*n* = 6)	2 (*n* = 8)	CBJE19, GBGJ22

Abbreviation: PRRSV, porcine reproductive and respiratory syndrome virus.

**Table 3:** Death in the PRRSV-infected groups.

Lineage	Strain	Mortality	Lung lesions in the dead pig
L1C	JB15-N-P31-GB	0/8 (0.00%)	—
	JB15-N-PJ73-GN	1/8 (12.5%)	Severe congestion, hemorrhage, pleuritis, bronchopneumonia

L5	VR2332	0/8 (0.00%)	—
	JB15-N-M8-GN	0/8 (0.00%)	—
	JB15-N-PJ4-GN	0/8 (0.00%)	—
	CNCY42	0/8 (0.00%)	—

LKA	CBJE19	0/8 (0.00%)	—
	GBGJ22	1/8 (12.5%)	Pleuropneumonia

LKB	GGYC45	2/8 (25.0%)	Edema, congestion, pleuropneumonia
	JB15-N-PJ10-GN	8/8 (100%*⁣*^*∗*^)	Severe bronchopneumonia, pleuritis, congestion

LKC	JB15-N-PJ45-GN	1/8 (12.5%)	Edema, bronchopneumonia

Abbreviations: L1C, lineage 1C; L5, lineage 5; LKA, lineage KOR A; LKB, lineage KOR B; LKC, lineage KOR C; PRRSV, porcine reproductive and respiratory syndrome virus.

*⁣* ^*∗*^In the JB15-N-PJ10-GN infected group, all remaining pigs were euthanized due to lethergy.

## Data Availability

The data in support of the findings of this study are available from the corresponding author upon reasonable request.

## Supporting Information

Supporting Information Figure S1. Maximum likelihood phylogenetic tree of global and Korean PRRSV-2 strains based on the ORF5 gene. The tree was constructed by RAxML-NG with 1000 bootstrap replicates using the GTRGAMMA nucleotide substitution model and the dataset of all global PRRSV-2 sequences available in GenBank subsampled by CD-HIT with a similarity threshold of 95%, as well as all Korean PRRSV-2 sequences available in GenBank as of Nov 2022. The color of the line in the phylogenetic tree indicates the lineage to which PRRSV belongs. The color of the circle on the phylogenetic tree indicates which country isolated it. Pink stars indicate the PRRSV strains used for challenge in this study. Yellow rhombuses indicate the vaccine strains.
