# Supplementary figures and images for: Pathogenic Characteristics of Five Different Lineage of Korean PRRSV-2 Isolates (NADC30-Like, VR2332-Like, LKA, LKB, and LKC)
Source: Transbound Emerg Dis. 2024 Oct 25;2024:1618472. doi: 10.1155/2024/1618472 (PMC12017120; doi:10.1155/2024/1618472)

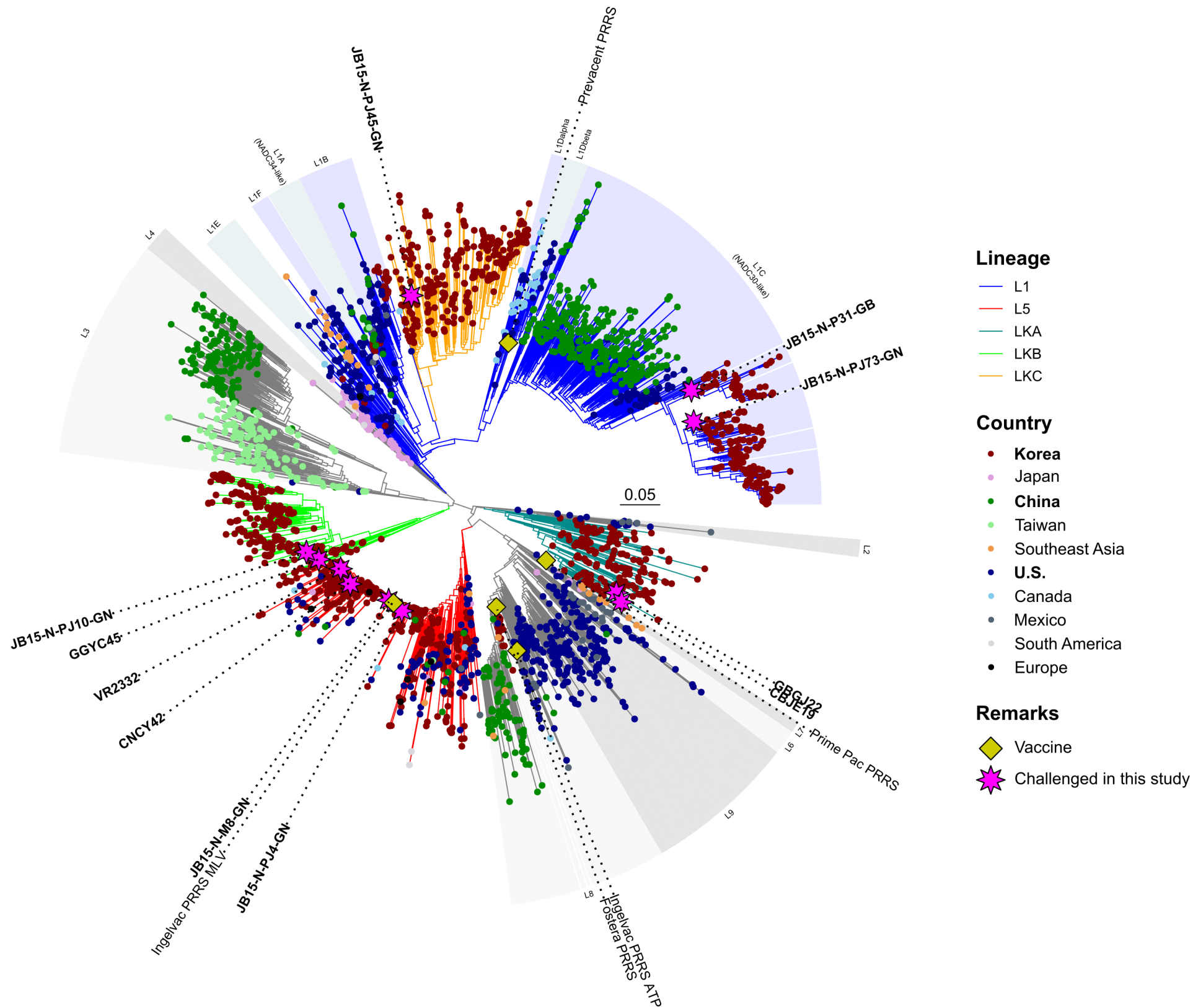

Supplement: Supporting Information — Figure S1. Maximum likelihood phylogenetic tree of global and Korean PRRSV-2 strains based on the ORF5 gene. The tree was constructed by RAxML-NG with 1000 bootstrap replicates using the GTRGAMMA nucleotide substitution model and the dataset of all global PRRSV-2 sequences available in GenBank subsampled by CD-HIT with a similarity threshold of 95%, as well as all Korean PRRSV-2 sequences available in GenBank as of Nov 2022. The color of the line in the phylogenetic tree indicates the lineage to which PRRSV belongs. The color of the circle on the phylogenetic tree indicates which country isolated it. Pink stars indicate the PRRSV strains used for challenge in this study. Yellow rhombuses indicate the vaccine strains. [file 1618472.f1.pdf]
